# Pulp regeneration with hemostatic matrices as a scaffold in an immature tooth minipig model

**DOI:** 10.1038/s41598-020-69437-6

**Published:** 2020-07-27

**Authors:** Ji-Hyun Jang, Joung-Ho Moon, Sahng Gyoon Kim, Sun-Young Kim

**Affiliations:** 1 Department of Conservative Dentistry, School of Dentistry, Kyung Hee University, Seoul, Korea; 2 Department of Conservative Dentistry and Dental Research Institute, School of Dentistry, Seoul National University, 101 Daehakno, Jongno-gu, Seoul, 03080 Korea; 3 Division of Endodontics, College of Dental Medicine, Columbia University, New York, NY USA

**Keywords:** Pulpitis, Pulp conservation, Root canal treatment, Pulp conservation, Root canal treatment

## Abstract

Control of blood clotting in root canal systems is one of the most critical and difficult concerns for regenerative endodontics therapy (RET). The purpose of this study was to investigate the effects of using gelatin- and fibrin-based hemostatic hydrogels as a scaffold on pulp regeneration in a minipig model*.* Cell viability of human dental pulp stem cells cultured three-dimensionally in gelatin-based and fibrin-based scaffolds was evaluated by MTT and live/dead assay. RET was performed on 24 immature premolars with an autologous blood clot (PC), gelatin-based and fibrin-based hemostatic matrices (GM and FM), or without the insertion of a scaffold (NC). The follow-up period was 12 weeks. Radiographic and histologic assessments for pulp regeneration were performed. Gelatin-based scaffolds exhibited significantly higher cell viability than fibrin-based scaffolds after 15 days (*P* < 0.05). The PC and GM groups showed favorable root development without inflammation and newly mineralized tissue deposited in the root canal system, while FM group presented inflammatory changes with the continuation of root development. The NC group exhibited internal root resorption with periapical lesions. The application of GM in RET led to favorable clinical outcomes of root development without inflammatory changes compared to conventional RET. Our results suggest that GM may serve as a viable regenerative scaffold for pulp regeneration.

## Introduction

Regenerative endodontics has been proposed to reconstruct the pulp-dentin complex through the application of the concept of tissue engineering^1,2^. The interplay of the tissue engineering triad, including cells, signaling molecules, and scaffolds, is essential for recapitulation of the biological events of tissue regeneration^3^. These three elements have been used either separately or in combination for the reconstitution of the pulp-dentin complex^4,5^. Cell-based therapy using exogenous stem cells has shown efficacy in regenerating pulp-like tissue in animal models^6–8^. However, it suffers in clinical translation due to difficulty of stem cell isolation and ex vivo cell expansion, risk of immune rejection and pathogen transmission, requirements for Good Manufacturing Practice facilities, and regulatory and economic barriers^2,4,5^. In contrast, the cell-homing strategy is used to regenerate the pulp-dentin complex with endogenous stem cells recruited into the root canal system and is more clinically translatable by circumventing many hurdles associated with the cell-based approach^2,4,5^. Indeed, a clinical procedure, “pulpal regeneration” adopted by the American Dental Association in 2011^9^, is based on the concept of the cell-homing approach to recruiting stem/progenitor cells by evoked bleeding.

Scaffolds serve as biological and structural supports for cell growth and differentiation to regenerate the desired tissue^2,3^. The development and selection of appropriate scaffolds conducive to pulp-dentin regeneration are one of the challenges in regenerative endodontics^2^. For pulp regeneration, an injectable gel-type scaffold is preferable because it adapts well to irregular root canal geometries and root canal intricacies to allow efficient cell–matrix interaction and controlled delivery of bioactive molecules^10^. Among the many injectable scaffolds, natural biodegradable polymers such as gelatin and fibrin have been widely used for tissue regeneration^11–14^.

Gelatin is a biopolymer protein derived from collagen hydrolysis^15^ and has been shown to facilitate proliferation and odontoblastic differentiation of dental pulp stem cells (DPSCs) in vitro^16^ and de novo vascular formation in vivo^11^. It exhibits better biocompatibility than collagen scaffolds due to its low immunogenicity^15^. Fibrin is another natural polymer protein involved in blood clot formation, which provides an initial extracellular framework for cellular activities for wound healing and tissue regeneration^12^. It has been demonstrated that fibrin-based hydrogels can promote pulp-like tissue formation with odontoblast layers in root canal systems in a rat model^14^. Gelatin and fibrin are used as commercial hemostatic matrix products through the design of a device to be mixed with thrombin in clinical medicine and dentistry. The pulp regeneration protocol by the American Association of Endodontics (AAE) contains a blood clot formation procedure for natural scaffold effects^17^ that comprises waiting until the event occurs. Clinically, waiting for blood clot formation is a rather time-consuming process, and the location of blood clot formation is uncontrolled even with the goal of having the entire canal system filled with blood to the level of the cementoenamel junction. Gelatin and fibrin, as forms of hemostatic matrices, may facilitate control of the time and location of blood clot formation in the root canal system and using the functions of tissue engineering scaffold recognized in the literature, and the pulp regeneration procedure can be more effective and efficient with the matrices. To our knowledge, this conceptual approach has not been performed in any animal model and thus requires validation.

Clinical studies using different types of scaffolds have not shown robust pulp-dentin regeneration^18–21^, even though regeneration of the pulp-dentin complex has been achieved in several animal studies using the cell-based approach^6,7,22–24^. Use of the cell-homing strategy with commercially available, biocompatible scaffolds may accelerate the clinical translatability of preclinical studies for reconstruction of the pulp-dentin complex. Therefore, the aim of this study was to investigate the effectiveness of commercially available gelatin-based and fibrin-based hemostatic matrix hydrogels on pulp-dentin regeneration in a minipig model with immature teeth. The histological outcomes of the cell-homing approach with blood clots used for clinical pulp regeneration were compared with those of a natural biopolymer hydrogel-based cell-homing strategy. The effect of scaffolds on DPSCs was also investigated by a cell viability assay in vitro*.*

## Materials and methods

### Cell preparation for in vitro testing

The study protocol using human permanent third molars (n = 3) was approved by the Institutional Review Board of Seoul National University Dental Hospital (IRB No. CRI19007) and was conducted in accordance with the guidelines and regulations of the Declaration of Helsinki. Third molars were extracted from donors (21 to 28 years old) for orthodontic and prophylactic reasons with informed consent. After tooth extraction, dental pulp was obtained and subjected to collagenase (900 u/mL) and dispase (400 u/mL) digestion at 37 °C for 1 h. Primary dental pulp cell cultures were performed with MEM Alpha (Invitrogen Corporation, CA, USA) supplemented with 15% (v/v) fetal bovine serum, 100 U/mL penicillin, and 100 μg/mL streptomycin at 37 °C with 5% CO_2_. The culture medium was changed every three days and sub-cultured at 70% confluence. At passage 2, cells were subjected to magnetic activated cell sorting (MACS) to obtain mesenchymal stem cells. For MACS, purified anti-human STRO-1 mouse IgM (BioLegend, San Diego, CA, USA) was used as the primary antibody, and anti-mouse IgM Micro Beads (Miltenyi Biotec GmbH, Bergisch Gladbach, Germany) were used as the secondary antibody. The sorting procedure was performed twice to obtain reliable stem cell fractions. Sorted cells at passage 4 were used for in vitro cell seeding.

### In vitro cell seeding

For the gelatin-based matrix scaffold (GM), 100 μL of gelatin solution was prepared with gelatin from porcine skin (Sigma-Aldrich, MO, USA) for each well of a 96-well plate. Then, 1 × 10^5^ DPSCs were seeded to the prepared gelatin solution, and 100 μL of thrombin solution from GM (FLOSEAL Hemostatic Matrix, Baxter Healthcare Corporation, CA, USA) was added and carefully mixed by gentle pipetting to prevent bubbles. After gelation, 200 μL of gelatin scaffold (gel strength = 300 g) containing 1 × 10^5^ DPSCs was formed for each well of the 96-well plate.

For the fibrin-based matrix scaffold (FM), fibrinogen and thrombin solutions were separated in a Greenplast-Q (Green Cross, Seoul, Korea) pre-filled syringe. Next, 100 μL of fibrinogen solution containing 1 × 10^5^ DPSCs was added to each well of the 96-well plate, and 100 μL thrombin solution was added and carefully mixed by gentle pipetting to prevent bubbles. After gelation, 200 μL of fibrin scaffold containing 1 × 10^5^ DPSCs was added to each well of the 96-well plate. The components of the matrix materials are shown in Table 1.

**Table 1:** Experimental groups of the in vivo study.

Group	Pulp Treatment	Scaffold matrix	Component of matrix material
Group NC	Pulp extirpation only	None	None
Group PC	Pulp regeneration	Intrinsic blood clot	None
Group GM	Pulp regeneration	Extrinsic synthetic gelatin-based matrix (Floseal, Baxter, CA, USA)	Purified bovine-derived gelatin with human-derived thrombin
Group FM	Pulp regeneration	Extrinsic synthetic fibrin-based matrix (Greenplast-Q, GreenCross, Seoul, Korea)	Human-derived fibrinogen with thrombin solution

*NC* negative control, *PC* positive control, *GM* gelatin-based matrix, *FM* fibrin-based matrix.

For control of in vitro cell viability testing and live/dead staining, 1 × 10^5^ DPSCs were seeded without scaffolds in each well of the 96-well plate. After cell seeding with and without scaffolds, 150 μL of culture medium was changed every three days and cultured at 37 °C with 5% CO_2_.

### MTT assay and live/dead staining

Viability of DPSCs in 3-dimensional cultures of GM and FM was assessed over 15 days using MTT assay. Briefly, after incubation of the scaffolds with 0.5 mg/mL of methylthiazolyldiphenyl-tetrazolium bromide (MTT, Sigma-Aldrich) for 60 min at 37 °C with 5% CO2, the converted dye was eluted with DMSO for 3 h. Absorbance was measured at λ = 570 nm. For each time point, measurement of cells without scaffolds served as a reference and was set to 100%.

To visualize live and dead cells, live/dead staining was performed on the 5th, 10th, and 15th days of in vitro cell culturing. A Live/Dead Viability/Cytotoxicity kit (Sigma-Aldrich) was used per the manufacturer’s instructions with slight modifications. Briefly, 10 μL of calcein-AM and 5 μL of propidium iodide solution were added to 5 mL of PBS to prepare the assay solution. The prepared scaffold containing the cells was washed with PBS 10 times to remove residual esterase activity, and 200 μL of assay solution was added to the scaffold and incubated at 37 °C for 30 min. Fluorescence was detected using an inverted fluorescence microscope (DS-Ri2, Nikon, Tokyo, Japan).

### Animal preparation

The in vivo study protocol was approved and performed in accordance with the guidelines of the Institutional Animal Care and Use Committee of Cronex Experimentation (Approval No. 2017-10001). A total of 24 immature premolars from four male minipigs (Cronex, Osong, Korea) aged 18 months, weighing approximately 90–95 kg was used. The minipigs were housed individually following standard laboratory conditions and fed a standard laboratory pellet diet and water ad libitum.

### Surgical procedure for pulp regeneration

The immature premolars of the four minipigs were divided into four groups of six teeth each. The groups and materials are presented in Table 1. The surgical procedures were performed under general and local anesthesia. Briefly, the animals were anesthetized by injecting 5 mL of 4:6 mixture of Rompun (5 mg/kg, BAYER), a muscle relaxant for animals, and the general anesthetic Zoletil (15 mg/kg, VIRBAC) in the ear vein. Anesthesia was maintained by aspirating a 2:1 gas mixture of isoflurane and oxygen during the surgery. Scandonest 3% plain (mepivacaine HCl 3%, Septodont, Seoul, Korea) was used for local anesthesia.

For teeth in the negative control (NC) group, the access cavity was prepared using a #330 carbide bur and a #02 round bur. Pulp extirpation and canal preparation were performed using stainless steel hand files and NiTi rotary files (Waveone gold, Large, Dentsply) with copious physiological saline irrigation. After canal drying using paper, the access cavity was restored with a 3 mm layer of chemically cured glass ionomer restorative material (Ketac molar aplicap, 3 M ESPE). For the positive control (PC) group, regenerative endodontic therapy (RET) was performed for the pulp extirpated tooth according to the AAE guidelines. Briefly, after pulp extirpation, the canal was prepared and irrigated with 10 mL of 1.5% sodium hypochlorite and 10 mL of 17% EDTA. Bleeding was induced through over-instrumentation of hand files 2 mm past the apical foramen so that the entire canal could be filled with blood to the level of the cementoenamel junction (CEJ). After blood clot formation, MTA (ProRoot MTA, Dentsply) was placed as a capping material, and a chemically cured glass ionomer restorative material was applied gently over the initially set MTA. For groups GM and FM, the same procedure was performed except that 0.6 mL of GM and FM were immediately applied in the root canal system after bleeding induction. After confirming hemostasis, MTA placement and glass ionomer restoration of the access cavity were completed.

### Radiographic and histologic assessments

Preoperative and postoperative periapical radiographs were taken to monitor the appropriacy of the clinical procedure. At 4- and 12-week follow-ups, periapical radiographs were taken under general anesthesia. Each periapical radiograph was blindly assessed by two examiners (SK and JJ) for evaluation of the presence or absence (yes/no) of periapical radiolucency, root resorption, progress of root development in length and width, and apical closure (n = 6).

At the termination of the experimental period at the 12-week follow-up, periapical radiographs were taken, and the minipigs were sacrificed through an anesthetic overdose of pentobarbital sodium. Block section samples of the jaws were dissected and fixed in 10% buffered formalin solution for histologic assessment. The samples were decalcified in 10% formic acid for eight weeks. Due to the difficulties in preparation and analysis of histologic samples with multi-rooted teeth, third premolars were excluded from histologic sample preparation and assessment (n = 3 ~ 6). Decalcified samples were embedded in paraffin blocks and serially sectioned longitudinally through the roots, followed by section staining with hematoxylin–eosin. Histology was assessed by two examiners (SK and JJ) for evaluation of the presence or absence (yes/no) of periapical inflammation, inflammatory cell infiltration, pulp-like tissue, tertiary dentin formation, and apex maturation.

### Statistical analysis

After the collected MTT assay data were assessed for Gaussian distribution by the Shapiro–Wilk test at a significance level of 0.05, an unpaired t-test was performed with the two-tailed model by GraphPad Prism version 8.3.0 for Windows (GraphPad Software, USA).

## Results

### In vitro cell viability

**Figure 1:**
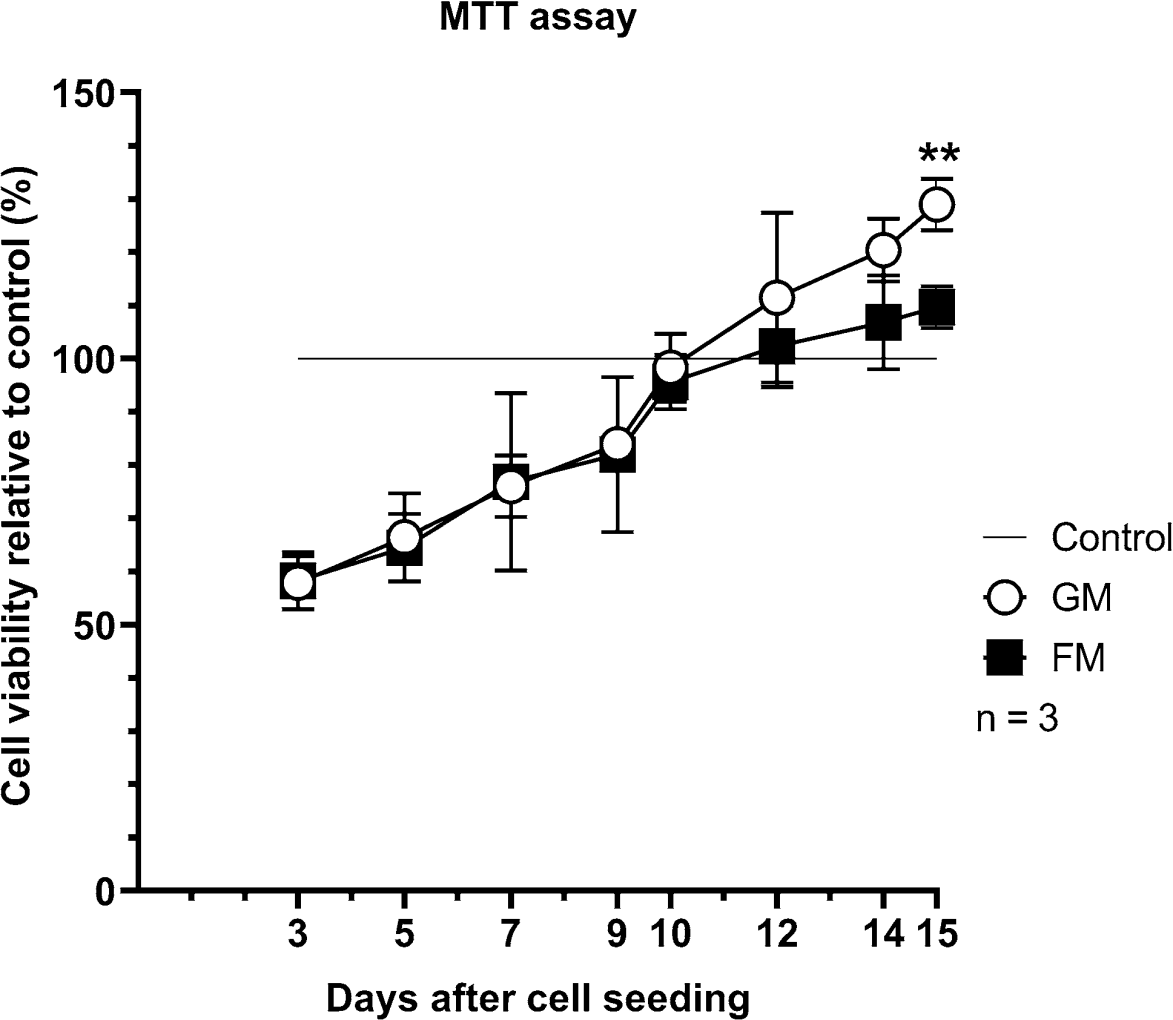
Cell viability of hDPSCs in gelatin-based and fibrin-based matrices. DPSCs were cultured in gelatin-based and fibrin-based three-dimensional (3D) scaffolds and in two-dimensional (2D) conditions without scaffolds as controls. For each time point, optical density measurements of cells in 2D conditions served as references and were set to 100%. On the 15th day of cell seeding, gelatin-based scaffolds exhibited significantly higher cell viability than fibrin-based scaffolds (*P* < 0.01, P = 0.0058). Three cell lines from three donors were used. **P < 0.01.

**Figure 2:**
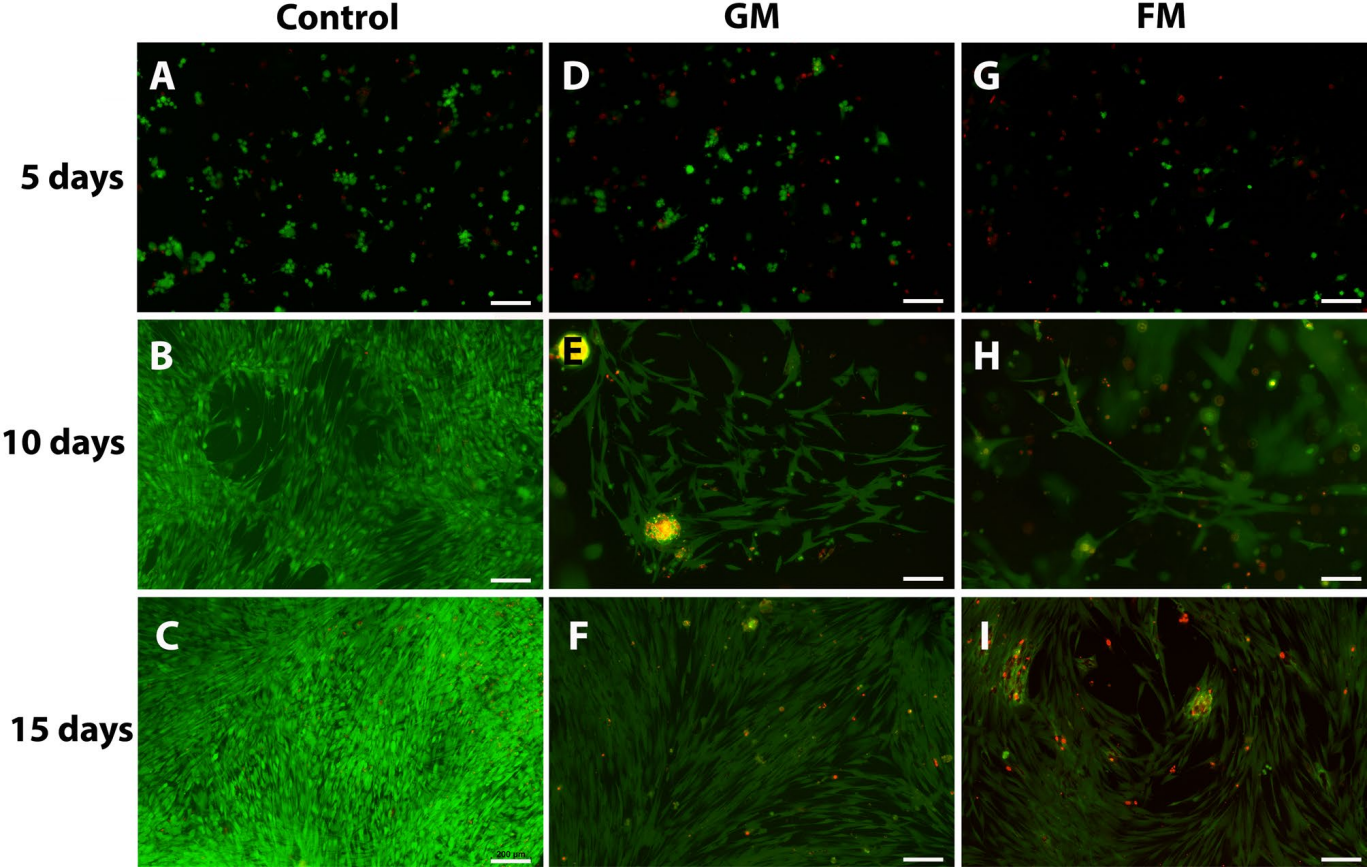
Live/Dead assay of hDPSCs in gelatin-based and fibrin-based scaffolds. DPSCs were cultured with gelatin-based (**D**–**F**) and fibrin-based matrices (**G**–**I**) in three-dimensional conditions and without scaffolds in two-dimensional conditions as controls (**A**–**C**). Green fluorescence indicates cytoplasm of live cells, while red fluorescence indicates nuclei of dead cells. Scale bar: 200 μm.

The MTT assay exhibited a generally similar growth profile of DPSCs in the GM and FM groups. However, cells cultured in the GM group had statistically significantly higher viability than those in the FM group on the 15th day of three-dimensional culturing (*p* < 0.05) (Fig. 1). Live/dead staining supported the observations from the MTT assays. The cell morphology and population appear to be similar between GM and FM. However, there were more cell-free spaces in FM compared to GM in longer-term culture periods (Fig. 2).

### In vivo outcomes for the pulp regeneration procedure

**Figure 3:**
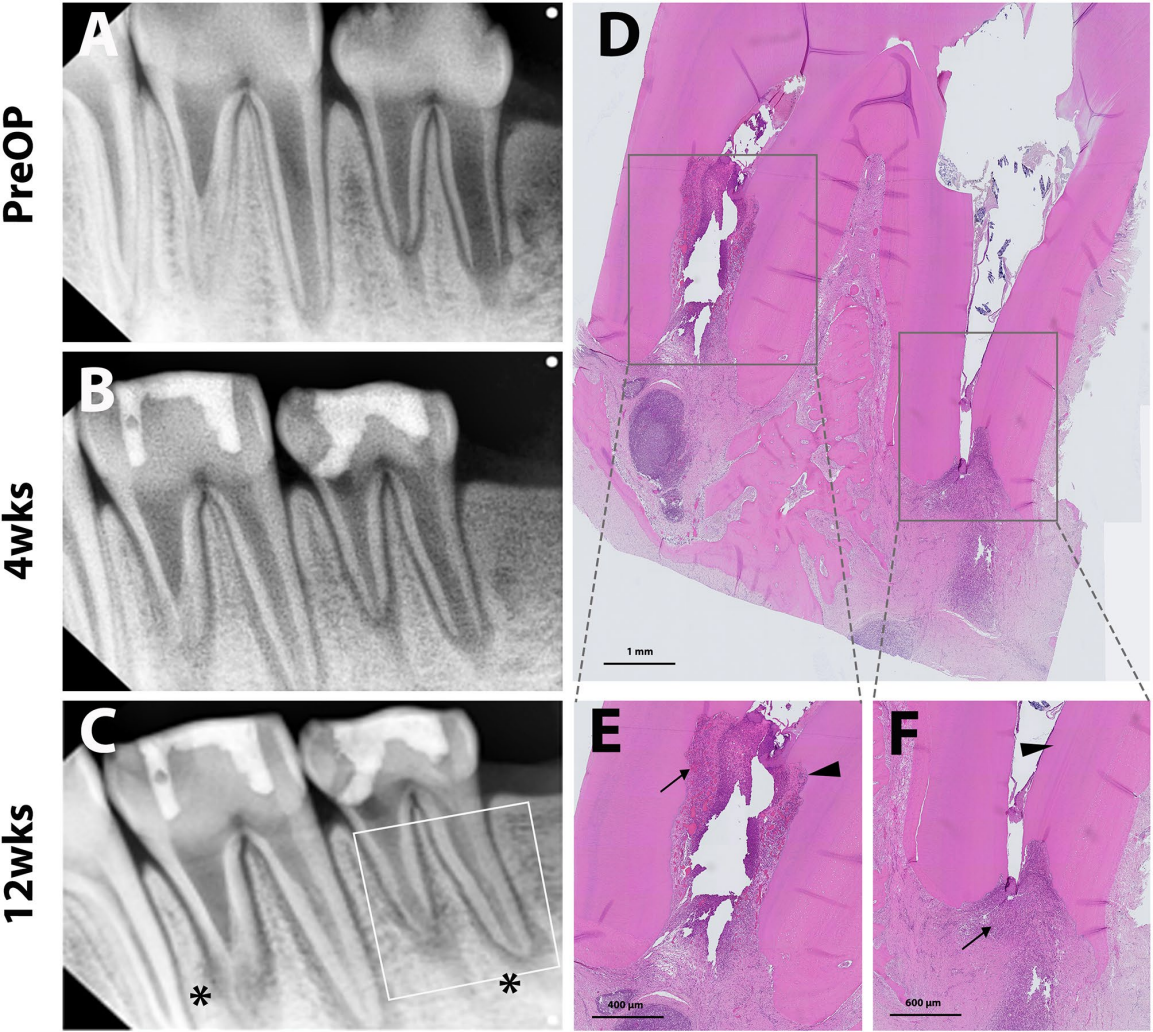
Representative radiographic and histologic images of the negative control (NC) group. Pulps were extirpated, and cavities were sealed directly with restorative glass ionomer cement. Periapical radiographs were taken preoperatively and at 4 and 12 weeks after treatment (**A**,**B**, and **C**, respectively). Internal root resorptive lesions were observed, which implicates pulpal inflammation, continuing root thickening/lengthening, and apical narrowing (white line square, **C**). Asterisks indicate the periapical radiolucency of the apices. After 12 weeks, the teeth were collected and processed for histologic analysis. Full images of the tooth showed periapical inflammation (**D**). Internal root resorption (arrowhead) with infiltration of inflammatory cells in the resorptive and periapical lesions (arrows) was observed (**E**). Newly-formed mineralized tissue deposition (arrowhead) in root dentin and inflammatory cell infiltration (arrow) in the apical area are shown (**F**).

**Figure 4:**
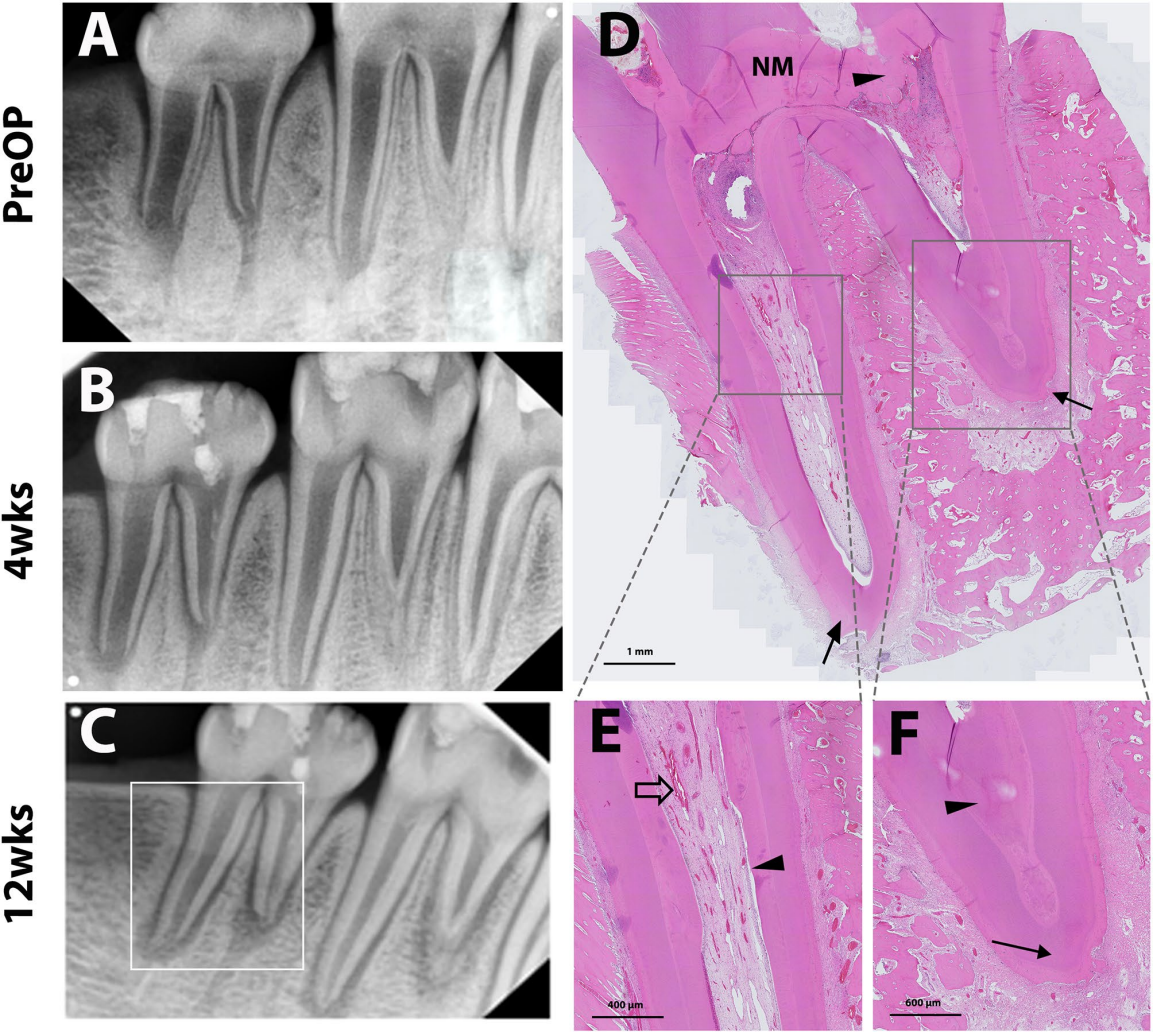
Representative radiographic and histologic images of the positive control (PC) group. Pulps were extirpated and regenerative endodontic procedures involving blood clot induction and MTA capping were performed, followed by restorative glass ionomer restoration. Periapical radiographs were taken preoperatively and 4 and 12 weeks after treatment (**A**,**B**, and **C**, respectively). Continuing root development without periapical radiolucency was observed (white line square, **C**). After 12 weeks, the teeth were retrieved and processed for histologic analysis. Full images of the tooth (indicated in the left periapical radiograph) showed newly-formed mineralized tissue (NM and arrowhead) adjacent to the MTA material and closure of the apices (**D**). A higher magnification image showed microvasculature (empty arrow) and an aligned odontoblastic layer (arrowhead) (**E**). Another higher magnification image showed newly-formed mineralized tissue (arrowhead) in root dentin without inflammatory cell infiltration in the apical area (arrow) (**F**).

**Figure 5:**
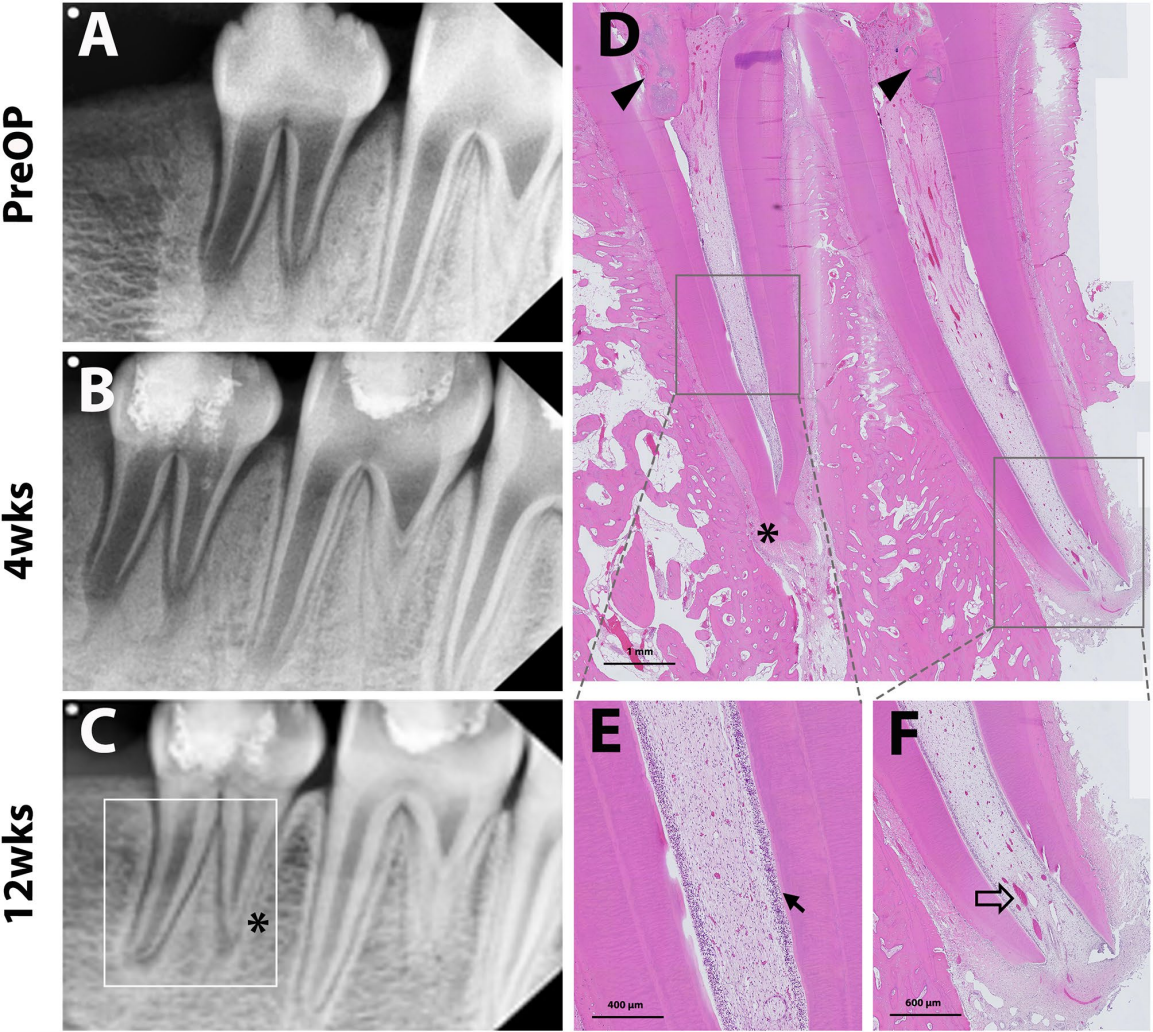
Representative radiographic and histologic images of the gelatin-based matrix (GM) group. Regenerative endodontic procedures were performed, involving GM application after bleeding induction, followed by MTA capping and glass ionomer restoration. Periapical radiographs were taken preoperatively and 4 and 12 weeks after treatment (**A**,**B**, and **C**, respectively). Continuing root development without periapical radiolucency was observed (white line square, **C**). After 12 weeks, the teeth were retrieved and processed for histologic analysis. Full images of the tooth (indicated in the left periapical radiograph) showed newly-formed mineralized tissue (NM, arrowhead) adjacent to the MTA material and closure of the apex of the distal root (asterisk) (**D**). Higher magnification images showed microvasculature (empty arrow) and well-organized odontoblastic layers (arrow) without inflammatory cell infiltration (**E**,**F**).

**Figure 6:**
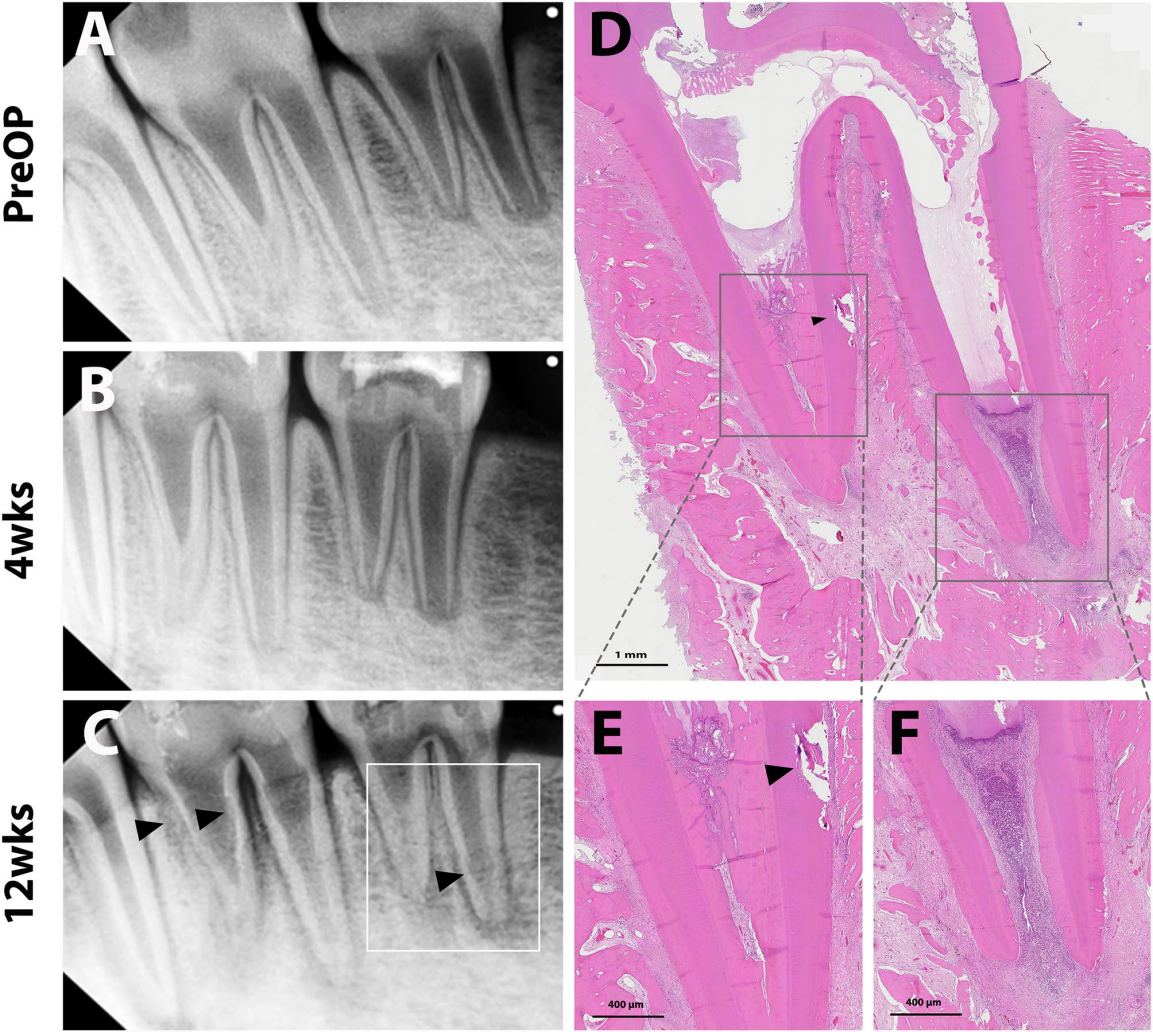
Representative radiographic and histologic images of the fibrin-based matrix scaffold (FM) group. Regenerative endodontic procedures were performed, involving FM application after bleeding induction, followed by MTA capping and glass ionomer restoration. Periapical radiographs were taken preoperatively and 4 and 12 weeks after treatment (**A**,**B**, and **C**, respectively). Internal root resorption (arrowhead) and periapical radiolucency in both roots were observed (white line square, **C**). After 12 weeks, the teeth were retrieved and processed for histologic analysis. Full images of the tooth (indicated in the left periapical radiograph) (**D**). Mesial root showing an irregularly obliterated root with newly deposited mineralized tissue without an odontoblastic layer (**E**), while the distal root showed a resorptive lesion with inflammatory cell infiltration (**F**).

The in vivo outcomes through the radiographic and histologic analysis of experimental groups are presented in Figs. 3, 4, 5, and 6 and Tables 2 and 3.

**Table 2:** Radiographic outcomes of the experimental groups after 12 weeks.

	NC (6)	PC (6)	GM (6)	FM(6)
Periapical radiolucency	100% (6)	17% (1)	0% (0)	67% (4)
Root resorption	50% (3)	0% (0)	0% (0)	50% (3)
Root thickening	83% (5)	100% (6)	100% (6)	83% (5)
Root lengthening	83% (5)	100% (6)	100% (6)	83% (5)
Apex closure	67% (4)	83% (5)	83% (5)	83% (5)

*NC* negative control, *PC* positive control, *GM* gelatin-based matrix, *FM* fibrin-based matrix.

**Table 3:** Histologic outcomes of the experimental groups after 12 weeks.

	NC (3)	PC (3)	GM (6)	FM(5)
Periapical inflammation	67% (2)	33% (1)	0% (0)	67% (3)
Inflammatory cell infiltration	100% (3)	0% (0)	0% (0)	60% (3)
Presence of pulp-like tissue	67% (2)	100% (3)	100% (3)	100% (5)
Newly mineralized tissue formation	67% (2)	100% (3)	100% (3)	100% (5)
Apex maturation	67% (2)	100% (3)	100% (3)	100% (5)

*NC* negative control, *PC* positive control, *GM* gelatin-based matrix, *FM* fibrin-based matrix.

In the 12-week follow-up radiographic analysis, NC and FM groups exhibited higher incidences of inflammatory changes, including periapical radiolucency and internal root resorption compared to the PC and GM groups. Specifically, GM showed the absence of periapical inflammation in any teeth. In the PC and GM groups, periapical radiographs revealed favorable root development in length and width without any inflammatory changes (Figs. 4 and 5). Interestingly, NC and FM groups also showed the root development indicated by continued root thickening, lengthening, and apex closure despite the inflammatory changes (Table 2).

In the histologic analysis, pulp-like tissues and deposition of newly formed tertiary dentin with apex maturation were observed in all the experimental teeth of the GM and PC groups. No intraradicular mineralized tissue deposition was identified in the GM group (Fig. 5), whereas part of the root canal was filled with the mineralized tissue in the PC group (Fig. 4). Higher magnification images showed microvasculature and odontoblastic layers aligned on the root dentin in GM and PC groups (Figs. 4 and 5). On the other hand, higher incidences of pulpal and periapical inflammation were shown in NC and FM groups. In those groups, the infiltration of inflammatory cells and the resorptive lesions with the presence of inflammatory cells were observed in the root canal system (Figs. 3 and 6). However, newly formed mineralized tissue deposition and apex maturation were presented in NC and FM groups despite inflammatory changes in the root canal system (Table 3).

## Discussion

Retention of stem cells at the target tissue with scaffolds is vital in regenerative tissue engineering. In the current procedure of RET, induction of intracanal bleeding, and formation of a blood clot acts as a regenerative scaffold^5^. This intrinsic natural scaffold forms a three-dimensional environment that provides a supportive structure for growth and differentiation of stem cells, leading to pulp regeneration. However, in clinical situations, it is quite challenging to control the appropriate blood volume and location of the blood clot in the root canal system through the injury of periradicular tissue. Even though a variety of scaffold materials has been used as endodontic tissue engineering scaffolds, including synthetic polymers such as polyglycolic acid (PGA), polylactic acid (PLA), and copolymer of PGA and PLA, poly(lactic-co-glycolic acid); natural polymers such as collagen, fibrin, and alginate materials and its derivatives; and self-assembling peptides^25^, these scaffolds do not meet all the essential characteristics of an ideal matrix scaffold for regeneration therapy—biocompatibility, biodegradability, resorption at a rate compatible with development of early regenerative tissue, and appropriate delivery convenience^26^. In this study, we investigated the possibility of using commercial hemostatic matrices—gelatin-based and fibrin-based—to immediately control bleeding in root canal systems when used as scaffolds in pulp regeneration procedures. Our results demonstrated improved in vitro cell viability in 3-dimensional cultures in the GM compared to the FM group*.* The GM group also presented favorable RET outcomes in in vivo experiments without any negative changes such as inflammation or resorption in the immature tooth minipig model at the 12-week follow-up. These results of the present study suggest that GM may act as a suitable regenerative scaffold material by controlling hemostasis in RET procedures.

Gelatin has the advantages of gel-forming, thickening, and emulsifying characteristics with favorable biocompatibility, mechanical properties, and biodegradation in normal healing time of 6 to 8 weeks^27,28^. Gelfoam is one of the most commonly used absorbable gelatin sponges for enhancing the healing of extraction wounds with favorable biocompatibility and cost-effectiveness. The use of a gelatin sponge was investigated in several studies on regenerative endodontics. Londero et al.^29^ and Wang et al.^30^ applied Gelfoam for RET procedures on immature molars and canines in dog models, respectively. Gelfoam, combined with intrinsic blood clots, significantly increased the formation of mineralized tissue along the dentin wall and presented favorable RET outcomes. In our study, we used GM as a gelatin-based regenerative matrix of an injectable commercialized hemostatic sealant (Floseal, Baxter) consisting of gelatin granules and a high concentration of human thrombin. The gelatin granules swell in the bleeding wound, producing a tamponade effect and enhancing initiation of the clotting cascade and clotting factors. Thrombin, another component of GM, converts fibrinogen into fibrin monomers, accelerating fibrin clot formation to initiate the coagulation cascade^27,31^. Our results showed that GM provided a mechanically stable matrix without hampering the biocompatibility of DPSCs in vitro (Figs. 1 and 2), which would be essential during the initial healing stage of pulp regeneration. Furthermore, GM is biodegraded within 6 to 8 weeks according to the manufacturer`s information, consistent with the timing of wound healing. This feature may have resulted in favorable radiographic and histologic outcomes with no inflammation or resorptive lesions in vivo, which is comparable to the PC group (Figs. 4 and 5).

A number of studies have reported that the use of platelet concentrates, such as platelet-rich plasma (PRP) and platelet-rich fibrin (PRF), improved biological regenerative outcomes compared to the use of conventional induced periapical blood clot^32–34^. Those platelet concentrates can form regenerative scaffolds through the fibrin mesh network to promote cell growth and differentiation^35^. Fibrin is a major structural blood clotting protein during the inflammatory process and plays a role in fibrinolysis, cellular and matrix interaction, and remodeling into the cell-derived extracellular matrix^36^. In this study, we used FM, a fibrin sealant (Greenplast-Q; GreenCross, Seoul, Korea), as the fibrin-based regenerative matrix. It is a commercialized two-component topical tissue adhesive hydrogel consisting of purified thrombin and fibrinogen. The use of fibrin sealant hydrogel, FM has been reported to be favorable in many biomedical fields for wound healing, vascular and neuronal anastomosis construction, and tissue engineering and regenerative medicine^36–38^. In the present study, FM exhibited a good outcome as a 3-D scaffold in the in vitro test showing increasing cell viability of DPSC over time similar to GM except for lower viability in longer-term cell cultures of 15th day. However, the in vivo outcomes of FM application in the root canal system showed a higher incidence of inflammation compared to the GM and PC groups. Even though our minipig model was pulpless and did not involve infectious necrotic pulp, approximately half of the experimental teeth revealed resorptive lesions on periapical radiographs (Table 3 and Fig. 6). The adverse consequences of FM may be related to the significant drawbacks of the substances, which include shrinkage of the gel itself or gel/cell construct and its rapid disintegration, resulting in loss of volume before the formation of adequate regenerative structures, as reported in previous studies^39,40^. It is assumed that contraction and rapid degradation of FM hydrogels formed a dead space in the root canal system and initiated defects, impeding the reparative and regenerative processes of the dental pulp. The lower cell viability in longer-term in vitro cultures of this study might be also related to the shrinkage and disintegration of substances over time. Several investigations have been performed to overcome this shrinkage limitation with composite scaffolds such as combining encapsulated stem cells in a fibrin gel with structural reinforced PGA mesh gels or sponge scaffolds^40,41^. The effectiveness of these adjusted fibrin gel scaffolds possessing controllable degradation properties on pulp regeneration must be investigated in future studies.

According to the latest AAE guideline, the primary goal of RET is the elimination of inflammatory symptoms and the resolution of periapical lesions, with the progression of root development in immature necrotic pulp teeth being secondary. An additional goal involves gaining functionally more organized, re-vitalized pulp tissue^17^. Conventionally, continued root maturation of immature teeth with residual inflammation or recurrent inflammation after RET was rarely possible because the primary goal failed to be achieved^42^. However, several recent case reports have shown that root maturation with immature teeth could be achieved even with persistent apical periodontitis in unsuccessful RETs, which is consistent with the findings in the present study^43,44^. In the current study, all teeth of the experimental groups exhibited newly mineralized tissue formation and apex maturation in the histologic analysis except for the NC group (Table 3). Radiographic outcomes also showed root development and signs of apical closure irrespective of inflammatory change in the root canal system (Table 2). Root development in failed RET teeth with inflammatory changes may result from the survival of resident or migrated stem cells to maintain their characterization despite persistent apical periodontitis. In addition, the specificity of immature teeth, which have powerful immune defenses and vascular innervation to respond to tissue injury, may allow the coexistence of root maturation and apical periodontitis^44^.

Miniature swine have been identified as a suitable animal biomedical model with the advantages of physiological, genetic, and anatomical similarities to humans^45,46^. In the dental regenerative field, minipig models have been utilized for various in vivo studies on pulp-dentin regeneration^46–48^, vital pulp therapy^49,50^, and periodontal bone tissue regeneration^51,52^. In this study, we used extirpated immature premolars of minipigs as a RET model to evaluate radiographic and histologic results over time. Due to the higher complexity of the root anatomy, volumetric size of the tooth, and difficulty of the operative approach, molars were excluded. However, the root anatomy of premolars was also challenging to analyze with periapical radiographs due to the bucco-lingual divergence of the mesial and/or distal roots. The palates of the minipigs were low in height and flat horizontally, creating difficulty in the positioning of the digital X-ray sensor around the maxillary premolars, resulting in variously elongated and distorted images. To minimize technical and projectional errors, extension cone paralleling (XCP) holders were used to approximately position the digital sensors in the middle of the palate. Another challenge in analyzing the minipig premolar model was the preparation for histologic samples, which included decalcification and sectioning due to the long and wide premolars and the large volume of the surrounding periodontal tissues. Our pilot experiment showed that 10% formic acid at room temperature for eight weeks was the appropriate decalcification condition with favorable histologic staining outcomes for minipigs.

Within the limitations of this study, we demonstrated that GM exhibits good cell viability of DPSCs in in vitro three-dimensional cultures and favorable RET outcomes with root development and no inflammatory changes in the immature teeth of a minipig model in vivo. The GM, consisting of gelatin granules and thrombin, may provide an adequate and stable 3-dimensional matrix with additional advantages of bleeding control in root canal systems without hampering the regenerative healing process. Our study suggests that a commercialized gelatin-based hemostatic matrix could serve as a viable regenerative endodontic scaffold for tissue engineering in regenerative endodontics.
